# Mechanical Vibration Measurement of Solidly Mounted Resonator in Fluid by Atomic Force Microscopy

**DOI:** 10.3390/mi8080244

**Published:** 2017-08-07

**Authors:** Fei Xu, Xinyi Guo, Linyan Xu, Xuexin Duan, Hao Zhang, Wei Pang, Xing Fu

**Affiliations:** State Key Lab of Precision Measuring Technology and Instruments, Tianjin University, Tianjin 300072, China; feifeixu@tju.edu.cn (F.X.); xinyiguo@tju.edu.cn (X.G.); xduan@tju.edu.cn (X.D.); haozhang@tju.edu.cn (H.Z.); weipang@tju.edu.cn (W.P.); xingfu@tju.edu.cn (X.F.)

**Keywords:** solidly mounted resonator, mechanical vibration, atomic force microscopy, amplitude modulation, lock-in amplifier

## Abstract

The very small vibration of a solidly-mounted resonator (SMR) in fluid may trigger a relatively large motion of the covering fluid, which was implied by our protein-related experimental results. Therefore, a series of experimental methods for characterizing the mechanical longitudinal vibration of the SMR and the corresponding out-of-plane dynamic response of the fluid above the SMR surface is described in this paper. A SMR device with theoretical resonance frequency of 2.5 GHz was driven by an amplitude-modulated (AM) signal, in which the amplitude is modulated by a signal of the second resonance frequency of the atomic force microscope (AFM) cantilever. A lock-in amplifier is used to demodulate the vibration response of the AFM cantilever, which is proportional to the amplitude of the sample vibration in contact mode and tapping mode. The amplitude-frequency curve of the SMR surface is obtained in contact mode with a relatively stronger interaction force between the AFM tip and the SMR surface. The amplitude-frequency curve of the motion of the liquid above the SMR device and the peak amplitude of the fluid at different distances above the SMR surface are measured in tapping mode with a relatively weak interaction force between the AFM tip and the fluid sample.

## 1. Introduction

In the last several years, film bulk acoustic resonator (FBAR) devices [1,2,3,4,5,6,7,8,9], especially the solidly-mounted resonator (SMR), has been increasingly studied as ultrasensitive biosensors [10], such as biosensors for human IgE detection [11], sensors for DNA hybridization detection, sensors for special protein detection, and immune sensors [12]. However, it is of vital importance to solve some issues concerning mechanical vibration characteristics of SMRs and the hydrodynamic responses of the fluid when the resonator is vibrating among these versatile applications. 

There is a considerable amount of literature investigating the vibration of various microresonators based on AFM. Ryder et al. presented a high-speed and accessible detection technique for an annular microresonator with contact-mode atomic force microscope (AFM) in air. The fundamental resonance mode was found to be 2.75 MHz with a quality factor of 6 and the peak amplitude is ~230 nm [13]. Two years later, a simple and direct method based on AFM operating in tapping mode was demonstrated to characterize the vibration amplitude of electrostatically-actuated MEMS resonators with the resonant frequency of 6.35 MHz and the corresponding amplitude of 90 nm [14]. The above two methods are suitable for a relatively stronger vibration, i.e., generally larger than 20 nm. The vibrations of the first three bending modes of carbon nanotube (CNT) resonators were mechanically detected in air via AFM. Especially for the third mode of CNT, the measured peak amplitude is only 0.02 nm at the resonant frequency 1.078 GHz. The CNT resonator is driven by an amplitude-modulated (AM) signal superposing on its eigenfrequency carrier signal, and the vibration response component of the AFM cantilever at an AM frequency, which is proportional to the vibration amplitude of the CNT resonator, could be extracted by a lock-in amplifier [15]. Similar experiments were carried out on suspended graphene resonators by Garcia-Sanchez [16]. In the same year, San Paulo et al. successively utilized atomic force microscopy in contact mode and dynamic force microscopy to characterize the mechanical vibrations of RF resonators including FBAR [17] in air. 

In our previous research, it was confusing that the proteins behaved with a much stronger dynamic response monitored by optical microscopy when motivated by the SMR device in fluid [18]. Consequently, the dynamic behavior of the bare SMR device without proteins was characterized using Garcia-Sanchez’s method [15]. It was found that even for the same SMR device, the resonant amplitude measured in fluid had a remarkable discrepancy with that measured in air. Thus, Garcia-Sanchez’s method has been comprehensively improved in this paper, which can obtain the SMR’s mechanical vibration properties, as well as the dynamic motion of liquid which is the direct surrounding environment of the proteins. The stiffness of SMR device, similar to the FBAR device [19], is much larger than CNT and graphene resonators. Here, contact mode, with a relatively stronger interaction force between the AFM tip and the SMR surface, and tapping mode, with a weaker interaction force, have been both applied to study the dynamic behavior of the SMR surface and the hydrodynamic motion of water induced by the vibration of the SMR. The measured amplitudes at different heights above the resonator surface have also been acquired to characterize the intensity of the movement of water.

## 2. Experimental Methods

### 2.1. Structure of the Solidly Mounted Resonator

**Figure 1:**
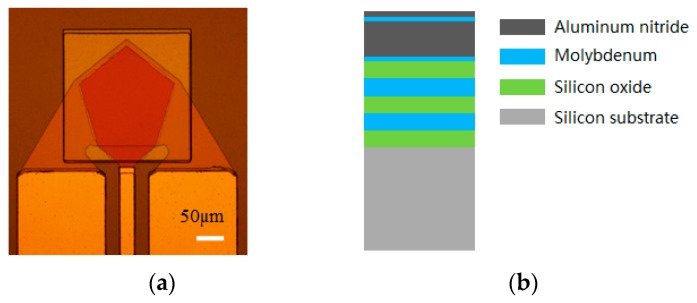
The schematic of SMR: (**a**) optical photo of SMR; and (**b**) cross-section of the pentagon region in (**a**).

Figure 1a,b illustrates the structure of the SMR used in this work. The resonant part of the SMR consists of a piezoelectric film of aluminum nitride (AlN) sandwiched between two molybdenum (Mo) electrodes (top and bottom). The overlapping region of the two electrodes defines a pentagonally-active area (0.02 mm^2^) of the SMR. The resonant part, which is more reliable than an air cavity in fluid, is isolated from the silicon substrate with a Bragg mirror consisting of silicon dioxide (SiO_2_) and molybdenum (Mo). Furthermore, a passivation layer composed of AlN is covered on the top electrode to protect the SMR from oxidation and corrosion. This device is designed to have a main thickness vibration mode at 2.5 GHz.

### 2.2. Experimental Setup and Principle

**Figure 2:**
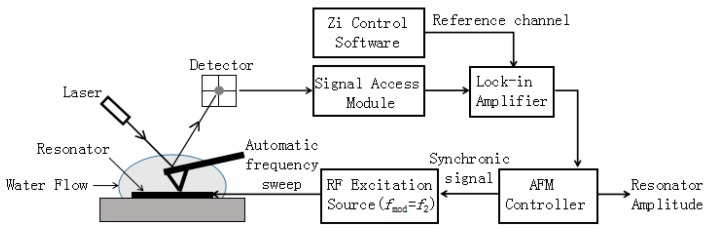
Automatic and fast experimental detection setup for contact/tapping mode of atomic force microscopy used in this work.

Figure 2 shows an integrated experimental setup used in this work. A commercial AFM (Dimension Icon, Bruker Corp., Bremen, Germany) can be operated in both contact mode and tapping mode. SNL-10 probes made of silicon nitride were purchased from Bruker Corp., and one of the cantilevers with a nominal spring constant of 0.35 N/m was selected. The AFM cantilever used is a triangular-shaped one with two arms. This cantilever has two clean peaks of resonant frequency. The first eigenmode frequency *f*_1_ and second eigenmode frequency *f*_2_ are ~18 kHz and ~110 kHz, respectively.

In Figure 2, the liquid covering the surface of the SMR is actually several drops of water placed on top of the resonator. The resonator is driven by a 100% amplitude-modulated signal with an RF power of 0 dBm provided by an RF excitation source, and the modulation frequency *f*_mod_ is equal to the second resonance frequency *f*_2_ of the AFM cantilever bending mode. As the AFM tip is scanning along the surface of the resonator, it follows the envelope of the resonator vibration and shows a response at the modulation frequency *f*_mod_ with an amplitude which is proportional to the resonator amplitude. The response of the AFM tip is transformed into electric signal through the detector. Afterwards, the magnified electric signal can be extracted by “signal access module (SAM)” and it then can be demodulated by the lock-in amplifier tuned at *f*_mod_, which is the same as the frequency of the reference signal set by the Zi control software. The demodulated signal is actually a voltage signal that can be displayed in the AFM software. In order to record the value of the signal automatically instead of an artificial reading, the AFM controller gives a “synchronic” signal to the trigger of the RF excitation source and the automatic frequency sweeps simultaneously. Finally, an input signal of the AFM laser photodetector in vertical direction is transferred into a voltage signal which corresponds to a resonator amplitude stored in AFM software. In tapping mode, the first eigenmode frequency *f*_1_ of the AFM cantilever should be used for topography imaging. Simultaneously, the second eigenmode frequency *f*_2_ is employed for the detection of the vibration amplitude with a lock-in amplifier tuned at *f*_mod_. Hence, it could be recognized as a “dual-frequency method”. Here, the AFM cantilever is sensitive to the longitudinal vibration, but is not sensitive to the shear mode vibration.

In contact mode, the value of deflection setpoint reflects different interaction forces between the AFM tip and the resonator surface. The larger the value is, the greater the interaction force increases. In tapping mode, tip offset is a parameter describing the distance between the AFM tip and the resonator within several micrometers. The value of the deflection setpoint and tip offset can be regulated at any time after the AFM tip is engaged. In order to demonstrate the influence of the water motion, a mapping of the amplitude versus deflection setpoint in contact mode and the amplitude versus tip offset above SMR surface in tapping mode are applied. 

## 3. Experimental Results and Analysis

### 3.1. Amplitude-Frequency Curve of SMR in Air

**Figure 3:**
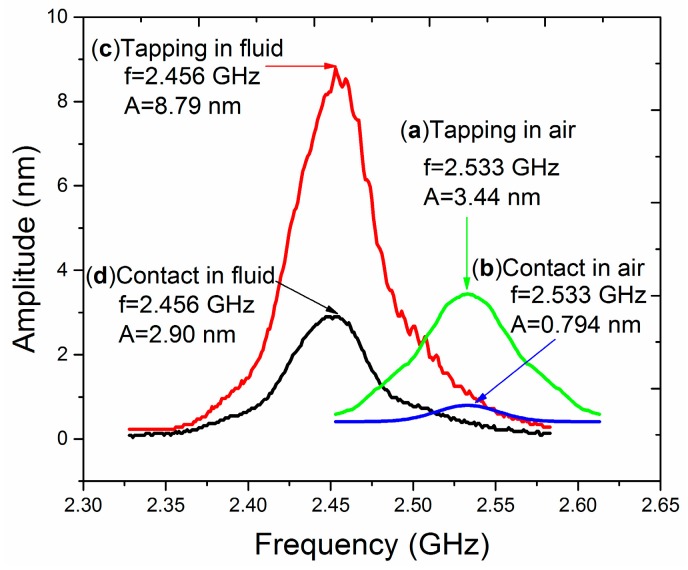
Amplitude-frequency curve measured in: (**a**) tapping mode in air; (**b**) contact mode in air; (**c**) tapping mode in fluid; and (**d**) contact mode in fluid.

Previously, our group used an SMR for driving biomolecules in fluid and found that the dynamic behavior of the particles was more intense than expected [20]. It was guessed that the phenomenon may be related to the fluid working environment. Therefore, we planned to measure the dynamic characteristics of the SMR operated in air and in fluid, respectively. First, the amplitude-frequency curve of the SMR working in air has been measured. According to Garcia-Sanchez’s method in tapping mode and contact mode in air, amplitude-frequency curves of the SMR driven by a 100% amplitude-modulated signal with an RF power of 0 dBm are shown in Figure 3a,b. It is easily seen from the curve that there is only one resonant peak at 2.533 GHz, and the amplitude is 3.44 nm in tapping mode and 0.794 nm in contact mode. The force exerted in tapping mode has little effect on the resonator vibration and the results obtained in tapping mode is closer to the real vibration amplitude of the SMR device [17,19].

### 3.2. Amplitude-Frequency Curve of SMR in Fluid

Utilizing identical measurement conditions and same radio-frequency power, the comparison test on the same resonator in Section 3.1 was carried on in fluid. Figure 3c shows an amplitude-frequency curve obtained in tapping mode in fluid. The free vibration amplitude of the AFM cantilever is set to be 300 mV, and the amplitude setpoint is set to be 150 mV, which is 50% of the free vibration amplitude. The range of sweep frequency is from 2.328 GHz to 2.583 GHz and the measured resonance frequency is 2.456 GHz. The resonance frequency of the resonator and Q-factors of amplitude-frequency curves are different in air and in fluid. The deviation of the resonance frequency reaches 77 MHz and the Q-factor differs from 63 in air (Figure 3a) to 58 in fluid (Figure 3c). The interaction of the SMR with liquid due to viscosity and the larger emission of acoustic power due to lower acoustic impedance mismatch are responsible for the frequency shift and the lower quality factor. 

In Figure 3c, the peak amplitude is 8.79 nm, which is ~2.5 times larger than the measurement result obtained in air in Figure 3a. Theoretically, the energy dissipation of the SMR in fluid is larger than that in air. It is reasonable to estimate that under the same power the vibration amplitude of SMR in fluid should be smaller than 3.44 nm (Figure 3a). The vibration amplitude of SMR in fluid should not be this large.

**Figure 4:**
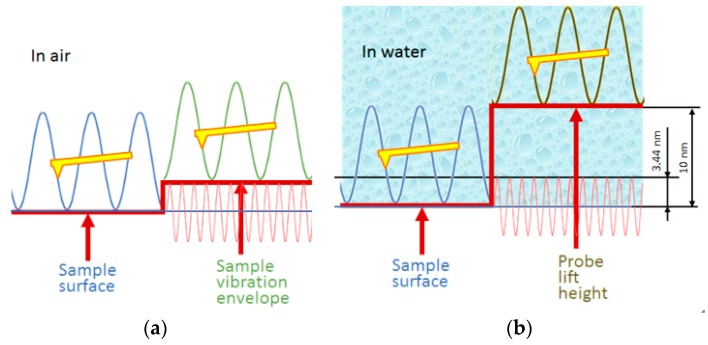
An illustration of the relative position of the cantilever and the sample: (**a**) in air; and (**b**) in water.

How can we verify that the amplitude detected is due to a large movement of the SMR surface or a dynamic influence of the liquid? A more straightforward experiment is conducted with a “single-frequency method” [14,21] in tapping mode in fluid. The idea of vibration amplitude measurement is shown in Figure 4a. When the SMR is not excited, let the probe tap on a single point of the SMR surface in imaging mode. The AFM cantilever will give out the position of the static sample surface as a reference. However when the SMR is excited by the RF signal without modulation, the AFM cantilever will step up to the envelope of the vibration peaks of the sample. The AFM cantilever acts as a mechanical low-pass filter as it encounters the RF high-frequency mechanical vibration of the SMR. Thu, it is easy to understand that the vibration amplitude of the SMR could be read from the step height in the topography of the scanning image in tapping mode. 

**Figure 5:**
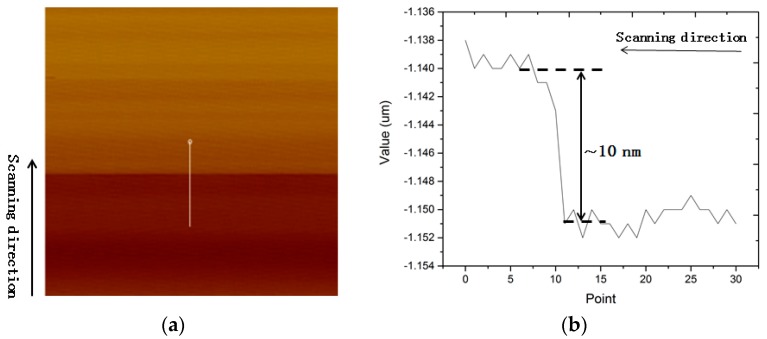
Height information of the AFM cantilever: (**a**) image of the height of the AFM cantilever with a half of excitation and another half without excitation on the SMR; and (**b**) values of the height sensor in the area where the white line in (**a**) lies.

Figure 5a shows the “step-type” topography of the static and vibrating SMR in fluid obtained from bottom to top. In Figure 5a, the upper half is recorded while the SMR is driven by a RF signal of 0 dBm. Figure 5b display the cross-section where the white line in Figure 5a is located. As is known from Figure 3a in Section 3.1, the vibration amplitude of the SMR with a RF signal of 0 dBm should be no larger than 3.44 nm. However, in Figure 4b, there is a ~10 nm step that the AFM cantilever lifts up sharply to a high position beyond the SMR surface. Figure 4b gives an illustration of the relative position of the cantilever and the sample. The vibration valleys of the cantilever do not reach the vibration peaks of the SMR. Thus, Figure 4 and Figure 5 indicate that the measured amplitude in Figure 3c in fluid in tapping mode is due to the hydrodynamic motion of the liquid above the SMR surface. Hence, the explanation for larger amplitude detected in tapping mode is that the mechanical vibration at the SMR surface can trigger much more intense movement of water in the vicinity. Consequently, the response of the AFM cantilever is not directly transmitted from the surface of SMR. The much stronger hydrodynamic motion of water hinders the contact of the tip and the SMR surface. As observed previously, the strong dynamic behavior of the proteins in fluid is reasonable to be considered the excitation of vibrating water [18].

**Figure 6:**
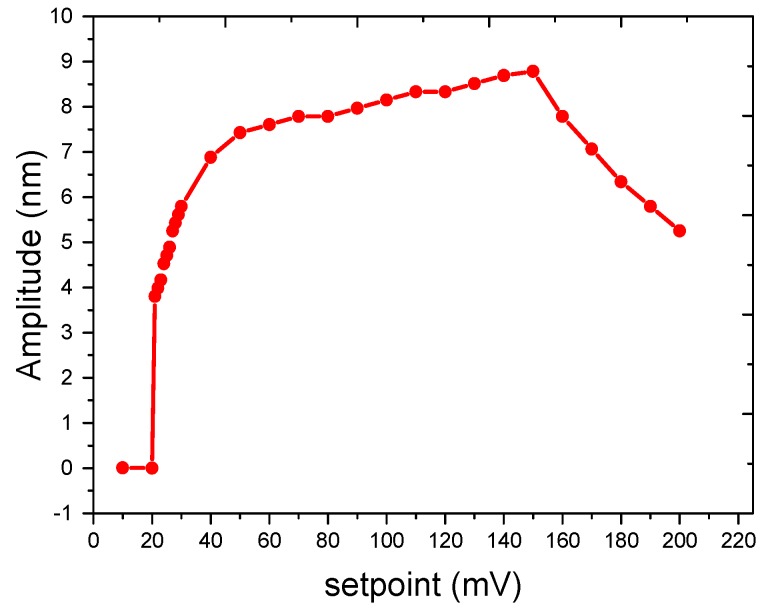
Amplitude response measured at different amplitude setpoints in tapping mode in fluid.

In order to obtain accurate and stable vibration amplitude of SMR surface, the idea of increasing the contact force between the AFM tip and the resonator surface has been attempted through changing the value of the amplitude setpoint in tapping mode. The relationship of the peak amplitude of SMR and the amplitude setpoint is shown in Figure 6, in which the measured vibration amplitude is commonly larger than that in air. The measured amplitude is larger than 8 nm when the amplitude setpoint is 33–50% (100–150 mV) of the free amplitude of AFM cantilever (300 mV). The measured amplitude is sharply damping to 0 when the amplitude setpoint is less than 7% (~20 mV). It is inferred that the AFM tip is easier to be influenced in fluid that the measurement method based on tapping mode is not suitable to detect the real vibration amplitude of the SMR device in fluid. Moreover, this kind of magnified effect was also found in other SMR devices working in fluid, and the ratio may sometimes be up to five times. Nevertheless, the unique application for the method is that it can be used to characterize the movement of water, which is just the working environment of our protein samples. In Section 3.3, the dynamic response of water at different height above the SMR surface will be further measured.

### 3.3. Measured Amplitudes at Different Heights above the Resonator in Tapping Mode

**Figure 7:**
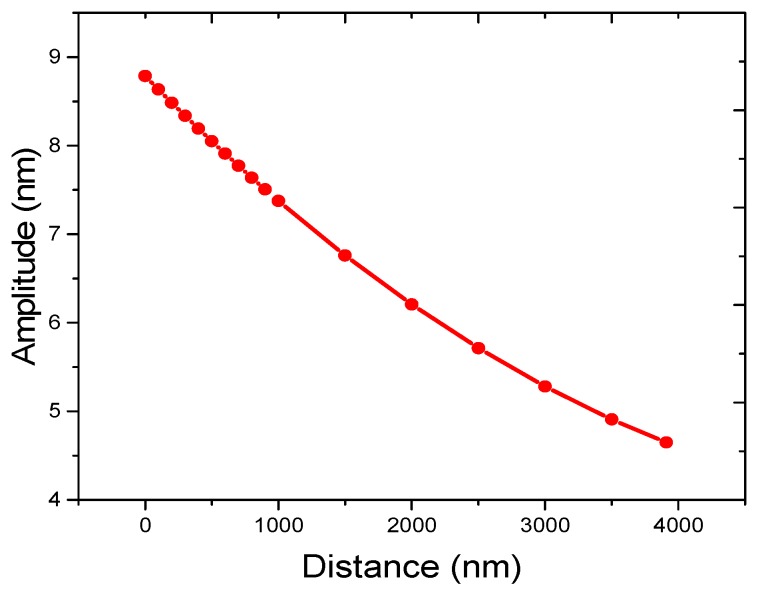
Amplitude versus different values of distance between the AFM tip and SMR.

According to Section 3.2, the motion of water has a significant effect on the measured amplitude. It is supposed that the magnitude of water motion differs with the height above the SMR surface. Through changing the values of tip offset, the distance between the AFM tip and the SMR surface can be adjusted. The corresponding measured amplitudes are shown in Figure 7. The distance ranges from 0 nm to 3.91 µm, and the reference position is the central position of the AFM tip working in Section 3.2. The corresponding measured amplitude gradually changes from 8.79 nm to 4.65 nm. It is inferred that the vibration intensity of water is negatively correlated with the distance above the SMR and the AFM tip experiences a relatively large impact force induced by the intense motion of the water. The method utilizing tapping mode is beneficial to investigate the movement of the water, and the results will help us significantly to understand the behavior of biomolecules.

### 3.4. Amplitude-Frequency Curve of SMR in Contact Mode in Fluid

Taking the weak interaction force in tapping mode into account, contact mode is better to measure the real vibration amplitude of the SMR regardless of the effect of water through setting a larger value of the deflection setpoint for a larger interaction force. Figure 3d shows an amplitude-frequency curve of the SMR in contact mode in fluid. The deflection setpoint is set to be 1.2 V. Considering that the spring constant of the AFM cantilever is 0.35 N/m and the sensitivity is 70 nm/V, the deflection setpoint value of 1 V amounts to a contact force of 29.4 nN between the AFM tip and the SMR. To compare with the result from tapping mode, the curve is obtained at the same position of the resonator with the same range of sweep frequency. By chance, the resonance frequency is also 2.456 GHz. The peak amplitude is 2.90 nm, which is slightly smaller than the data obtained in air. Here, caution must be taken while obtaining the curve. Only under the condition that the deflection setpoint is set to be large enough, the AFM tip can penetrate the water layer between them and sense the accurate vibration amplitude of the device. We assume that the choice of the interaction force is reasonable within a certain range and the confirmation of the specific parameters need further experiments, as described in Section 3.5. 

**Figure 8:**
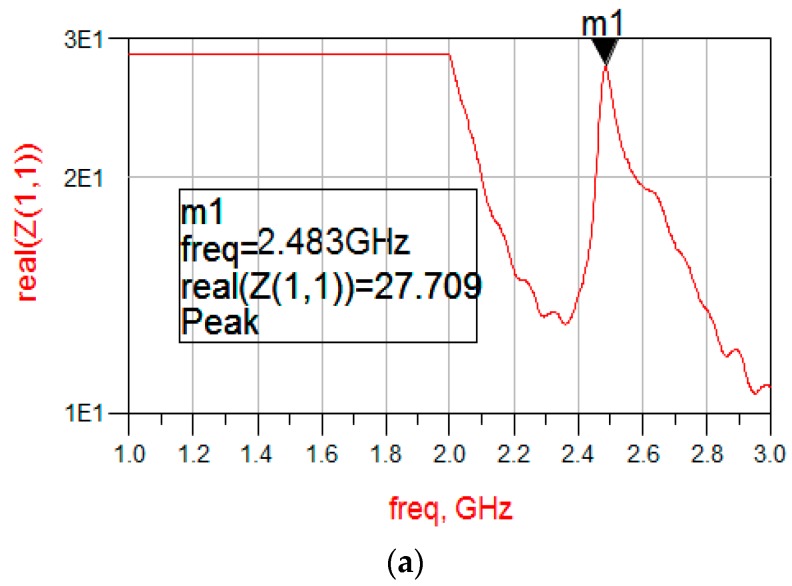

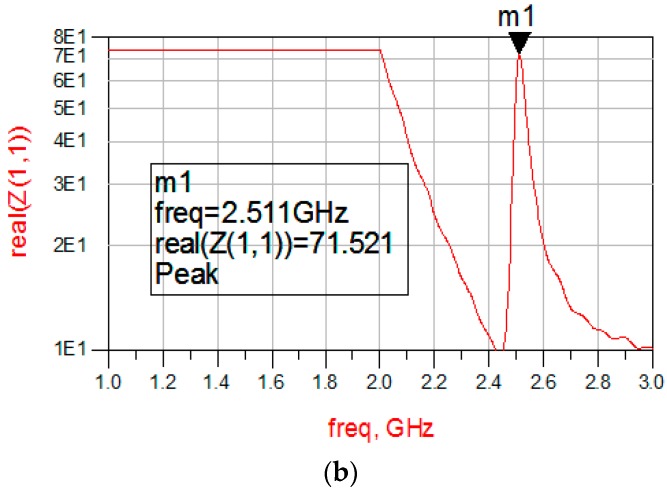
Frequency response curve of the SMR measured by a network analyzer: (**a**) in fluid; and (**b**) in air.

Additionally, in order to study the relationship of the mechanical resonance frequency of the SMR and electrical resonance frequency of the series-circuit model, the electrical characterization of the SMR is measured by the network analyzer. The electrical results are shown in Figure 8. It can be seen that the resonance peak is 2.483 GHz in fluid, which has only ~27 MHz deviation according to the result introduced above and the resonance peak increases to 2.511 GHz in air that has ~22 MHz less than that measured by AFM. The AFM method based on contact mode with proper parameters cannot only detect amplitude-frequency curve of SMR surface vibration, but also indicates the inner consistency of the electrical resonance frequency and the mechanical resonance frequency.

### 3.5. Measured Amplitude with Different Interaction Force between the AFM Tip and the Resonator in Contact Mode

**Figure 9:**
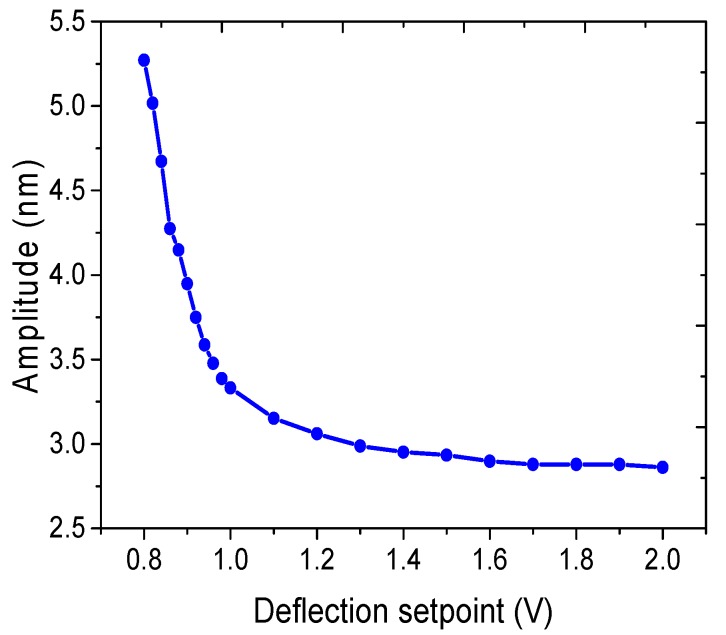
Amplitude response measured at different deflection setpoints in contact mode.

If the value of the deflection setpoint is set to be smaller, the interaction force between the AFM tip and the resonator will also become smaller. As a result, the measured amplitude will increase. The relationship between the measured amplitude of the SMR and the deflection setpoint is shown in Figure 9, in which the setpoint increases from 0.8 V to 1.0 V with 0.02 V intervals and from 1.0 V to 2.0 V with 0.1 V intervals in order to obtain a relatively smooth and continuous curve. As the value of the deflection setpoint increases from 0.8 V to 2.0 V, the vibration amplitude decreases from 5.27 nm to 2.90 nm. It can be seen that with the increasing deflection setpoint larger than 1.2 V, the amplitude tends to be a constant value of 2.90 nm. It is hypothesized that the interaction force between the AFM tip and the SMR surface contributes to this result. As the value of the deflection setpoint varies from 0.8 V to 1.2 V, the tip is more easily affected by water motion as a result of not contacting the resonator closely. Here, when the value of the deflection setpoint is set to be 1.2 V to 2.0 V, the measured amplitude will not be influenced by water. The SMR device used in this paper has a larger stiffness, which is determined by its structure. Thus, the larger force does not seriously affect its amplitude attenuation.

## 4. Conclusions

In summary, a comprehensive detection method based on atomic force microscopy in contact mode should be employed for the sake of obtaining the real longitudinal vibration amplitude of a solidly-mounted resonator in fluid. It is noted that there should be a relatively large interaction force between the AFM tip and the resonator. In addition, a series of improved methods in tapping mode utilizing a frequency sweep and tip offset change are suitable to measure the out-of-plane vibration amplitude caused by the motion of water. Differently-measured amplitudes can reflect different intensities of the movement of water. With respect to the SMR, the vibration amplitude of its surface in fluid is slightly smaller than that in air. In a fluid environment, there exists a relatively intense longitudinal vibration of water covering the surface of the SMR. The frequency response characteristic of the water above the SMR surface is similar to that of the SMR itself, and the maximum value of the amplitude occurs close to the surface of the SMR. The vibration amplitude decreases with the increased distance away from the SMR surface. According to incomplete statistics, the peak longitudinal vibration amplitude of water is 2–5 times than that of the SMR. Consequently, the method also provides a new thought to explain the dynamic behavior of protein above the working SMR device in fluid.
